# The Effect of Metal-Semiconductor Contact on the Transient Photovoltaic Characteristic of HgCdTe PV Detector

**DOI:** 10.1155/2013/213091

**Published:** 2013-09-30

**Authors:** Haoyang Cui, Yongpeng Xu, Junjie Yang, Naiyun Tang, Zhong Tang

**Affiliations:** School of Electronic and Information Engineering, Shanghai University of Electric Power, 2103 Pingliang Road, Shanghai 200090, China

## Abstract

The transient photovoltaic (PV) characteristic of HgCdTe PV array is studied using an ultrafast laser. The photoresponse shows an apparent negative valley first, then it evolves into a positive peak. By employing a combined theoretical model of *pn* junction and Schottky potential, this photo-response polarity changing curves can be interpreted well. An obvious decreasing of ratio of negative valley to positive peak can be realized by limiting the illumination area of the array electrode. This shows that the photoelectric effect of Schottky barrier at metal-semiconductor (M/S) interface is suppressed, which will verify the correctness of the model. The characteristic parameters of transient photo-response induced from *p-n* junction and Schottky potential are extracted by fitting the response curve utilizing this model. It shows that the negative PV response induced by the Schottky barrier decreases the positive photovoltage generated by the *pn* junction.

## 1. Introduction

Compared with Quantum-Well-Infrared Photodetector (QWIP), HgCdTe Focal-Plane Array (FPAs) have the advantages of high detection rate, high response speed, and wide detection band and have been widely applied in aerospace infrared optical remote sensors, scientific satellites, military defense and meteorological observation [1–3]. To realize the conversion of optical signals into electrical signals, the photo carriers will be injected into the readout circuit through the HgCdTe-Metal interface in the basic pixel structure. This requires the electrode forming an Ohmic contact at the M/S interface [4]. Generally, the interface barrier layer will become very thin if the metal contact with the heavily doped *n* ^+^-HgCdTe, so that the carriers can tunnel through these barriers. Thus, M/S interface can form an Ohmic contact. But due to the fragile nature of the HgCdTe material, *p* HgCdTe is not easy to form Ohmic contacts with metal, and it is even possible to form a Schottky contact [5, 6]. Thus the *I*-*V* characteristics will be impacted by the rectified characteristic of the M/S interface electric field. The more obviously the Schottky junction effect is, the serious influence of *I*-*V* characteristic is likely to be suffered. Currently, this approach has become a common means to research interface contact characteristics and evaluate the quality of Ohmic electrodes [7–9]. Nevertheless, there are still some deficiencies using this method. Firstly, the interface information can only be extracted from the *I*-*V* characteristic when the Schottky junction effects become more serious. Secondly, the impact of Schottky junction effect on the PV conversion mechanism cannot be determined from *I*-*V* characteristic measurements. 

The transient photovoltage (TPA) measurement will lead to a variety of novel physical phenomena and a diversity of new phases which the conventional optical characterization methods are difficult to observe, and this will have a special advantage for the enrichment and development of semiconductor optical theory. With the ultrafast pulsed laser excitation, optical devices will generate a lot of photo-generated carriers, which is very sensitive to the internal electric field of the semiconductor; thus, the transient photovoltaic properties demonstrated will provide important clues about the source of discovering new features for optoelectronic devices. Generally, there is a big asymmetry of electric field in Schottky barrier at M/S interface with the *pn* junction in semiconductor devices, and the differences of the amplitude and frequency characteristics will exist inevitably. This will provide the possibility to extract the information from the TPV. In this reason, this paper will carry out the detailed studies the of PV response of HgCdTe PV array utilizing TPV. The experiment results show an apparent negative valley first, then it evolves into a positive peak. By employing a combined theoretical model of *pn* junction and Schottky potential, this photo-response polarity changing curves can be interpreted well. Due to the Schottky barrier of M/S interface, the negative PV response induced will decrease the positive signal generated by *pn* junction, and consequently reducing the response rate. Since the characteristics of *p-n* junction and Schottky barrier will interfere each other in the conventional *I*-*V* test, it is very difficult to assess the quality of the electrode. While the characteristic can be distinguished by exploiting the TPV, this method may have an advantage compared with the conventional electrode assessment methods, and the sensitivity is expected to be greatly improved.

## 2. Experiment

**Figure 1:**
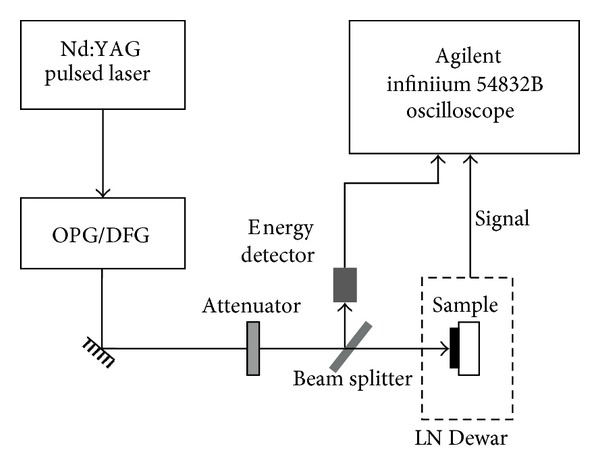
Schematic diagram of the transient photovoltage technology (not to the scale).

The HgCdTe planar *pn* junction PV detector was grown by MBE on GaAs substrate with a buffer layer of CdTe. The acceptor and donor concentration were 8 × 10^15^ and 1 × 10^17^ cm^−3^, respectively. The Cd composition was 0.298 (the cutoff wavelength is approaching 4.8 *μ*m). The junction area was 50 × 50 *μ*m^2^. The experimental system consisted of four major parts: liquid nitrogen (LN) Dewar, wavelength tunable ultrafast pulsed lasers, energy monitoring system, and digital Storage oscilloscope, which is shown in Figure 1 [10, 11]. The sample was mounted in LN Dewar for measurement, and the temperature was near 77 K [10]. The incident laser pulse was provided by a Picosecond Nd:YAG laser (EXSPLA PG401/DFG). The Laser delivered pulse of 30 ps in duration at a frequency 10 Hz. Comparing to the shortest rising time of tens of nanoseconds in the pulsed response profile of the HgCdTe photodiode, the 30 ps laser pulse can be approximated as a *δ* function in our experiment. A small portion of the laser beam was reflected by a beam splitter and measured using an energy detector in order to monitor the exciting energy. The pulsed photo-response of the HgCdTe detector was measured from the voltage drop across a 50 Ω load-resistor. Both signals from the energy detector and the HgCdTe detector were fed into an oscilloscope through BNC connectors to monitor and record the pulse profiles. An average of 200 pulsed profiles was recorded to eliminate the pulse-to-pulse fluctuation and improve the signal to noise ratio. An aperture was used to limit the illumination area of the linear array detectors by blocking the laser beam. 

## 3. Results and Discussion

**Figure 2:**
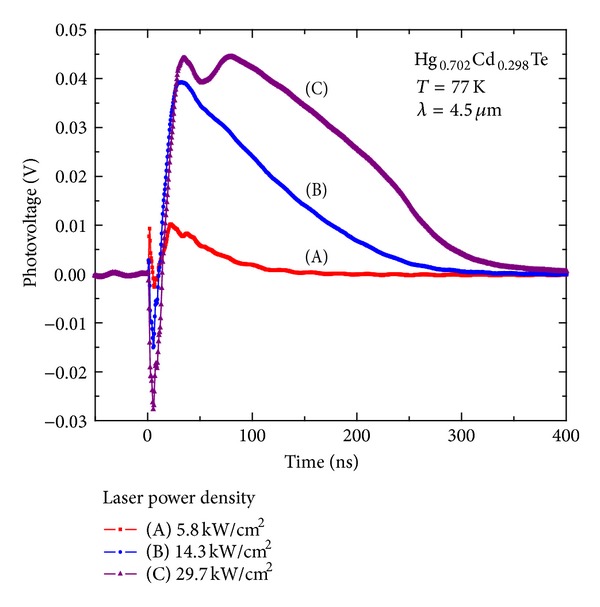
Transient PV response of the detector illuminated by the pulsed laser with the photon energy larger than the bandgap for the different incident intensity.

**Figure 3:**
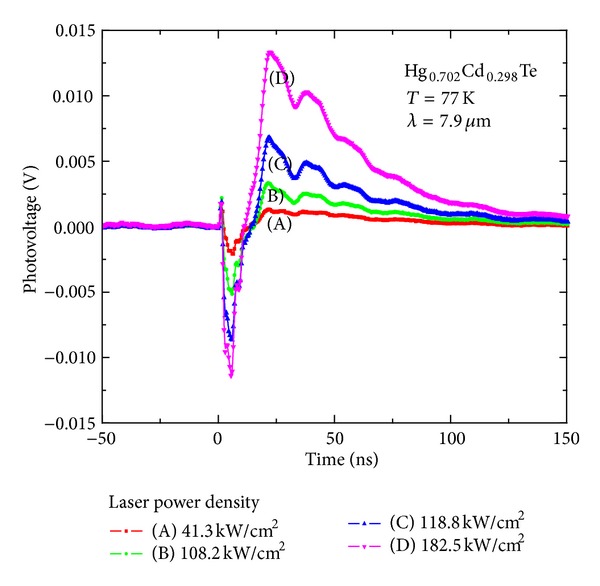
Transient PV response of the detector illuminated by the pulsed laser with the photon energy smaller than the bandgap for the different incident intensity.

The basic mechanism of *n* ^+^-on-*p *HgCdTe PV photodiode is the following: The incident photon absorbed by *p*-HgCdTe layer will generate photo carriers. These carriers will be separated by the build-in electric field and form the photovoltaic response. Therefore, the ideal PV response should show a rapid increase and slow decay process [10]. However, the TPV response time profiles of the HgCdTe photodiode shows an apparent negative valley during the first 15 ns, and then it evolves a positive peak. By changing the excitation laser intensity, the transient photo-response of the detector shows the similar time evolution profiles, no matter for the case of one-photon absorption (OPA) transition that the photon energy is larger than the bandgap (shown in Figure 2) or for the case of two-photon absorption (TPA) transition that the photon energy is smaller than the bandgap (shown in Figure 3). This shows that there is a new mechanism for the photo-response polarity changing, where the phenomenon has been observed before [11]. For subsequent discussion and data analysis, some experimental results are listed here.

The negative TPV of experiment result can be attributed to the Schottky barrier at the metal-HgCdTe interface. Because the *p*- and *n*-electrodes of the array are in the same plant, if a Schottky contact is formed at the interface of the metal layer with the *p*-HgCdTe surface, then it will constitute a class *n* ^+^-on-*p* junction. The opposite built-in electric field of the Schottky barrier contact comparing to *pn* junction provides the possibility for the generation of negative valley and positive peak in the TPV, while the high-frequency characteristic of the Schottky barrier [12] provide the possibility for the generation of negative PV prior to positive ones. 

**Figure 4:**
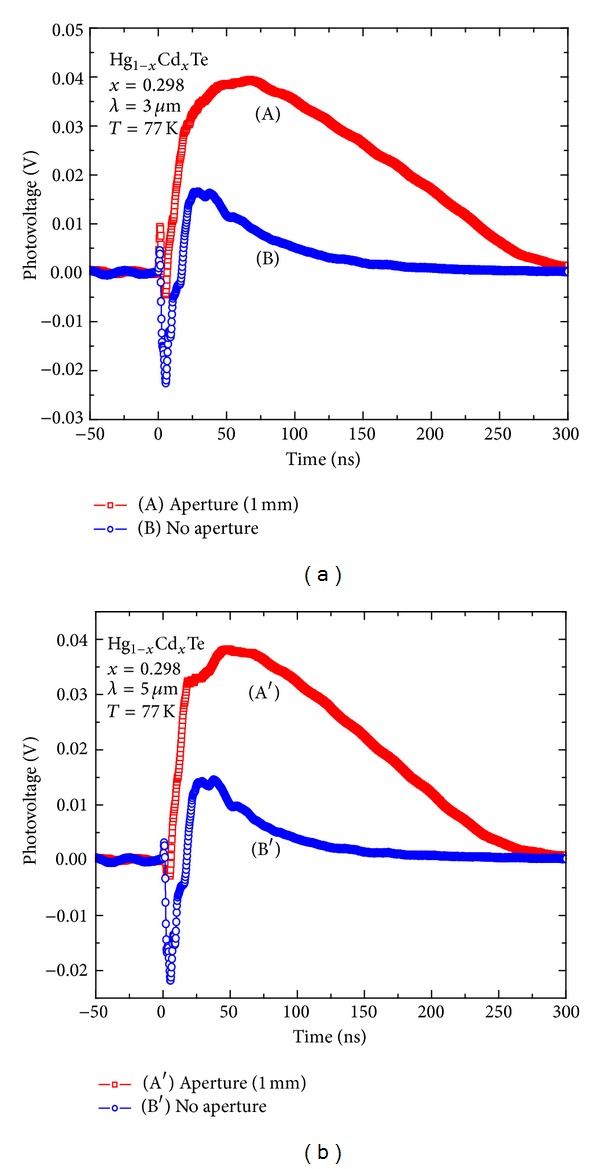
Transient PV response of the HgCdTe photodiode with no aperture and 1 mm aperture added in the optical path for incident photon energy larger and smaller than the bandgap.

The negative PV response derived from Schottky barrier mechanism of M/S interface can also be confirmed by the transient PV response experiment measurement with and without aperture added in the optical path for photon energy larger (OPA, shown in Figure 4(a)) and smaller (TPA, shown in Figure 4(b)) than the bandgap. For the array detector used in this experiment, the common *p*-electrode configurations surround all pixels in the linear array of the detectors. Because the size of pixel is only 2.5 × 10^3^ *μ*m^2^, the spot of the incident laser reaches 50 mm^2^, which is about 2 × 10^4^ times larger than the pixel area. Thus the *p*-electrode covering area will be illuminated inevitably and even will constitute a major part of the light receiving area if the incident laser beam has been not limited by aperture. If a Schottky contact is formed at the interface of the metal layer with the *p*-HgCdTe surface, the magnitude of the negative TPV generated from M/S interface should be sufficient compare with that of *pn* junctions. However, the negative PV valley will be suppressed, and the positive PV will be enhanced when the size of the incident laser beam is limited by aperture. Thus, the ratio of negative valley to positive peak can be realized by limiting the illumination area of the array electrode. The experimental results demonstrate that the PV response effect of Schottky barrier at M/S interface is suppressed if the illumination area of *p*-electrode interface is decreased. So the correctness of the combined theory model is verified.

From the discussion mentioned above, one can interpret this photo-response polarity changing TPV by employing a combined theoretical model of *pn* junction and Schottky potential. Since the Schottky barrier has the similar characteristic to *n* ^+^-on-*p* junction and the TPV curves of *pn* junction show the form of a typical pulse function, the apparent TPV curves of the detector can be expressed as a superposition of two single pulse function:

(1) V(t)=Vschottky(t)+Vpn(t)=ΔVschottky[1−exp⁡(−tτ1)]exp⁡(−tτ2) +ΔVpn[1−exp⁡(−tτ1′)]exp⁡(−tτ2′),

where Δ*V*_schottky_, *τ*_1_, and *τ*_2_ are the photovoltage, pulse rise time, and fall time of Schottky contact, respectively. Δ*V*_*pn*_, *τ*_1_′ and *τ*_2_′ are the photovoltage, pulse rise time and fall time of *pn* junction, respectively.

**Figure 5:**
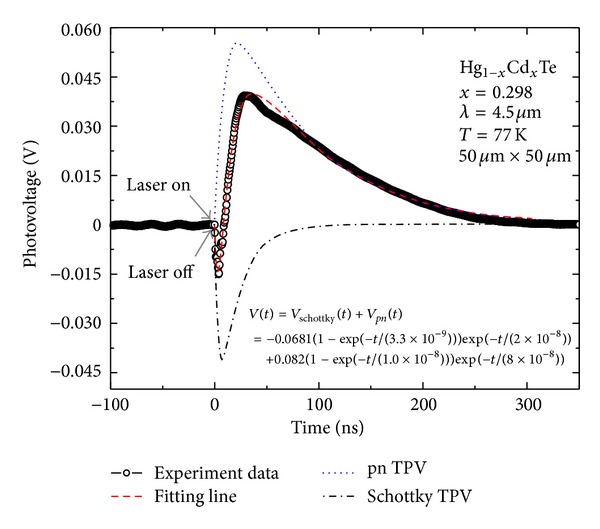
Pulsed PV response profiles from HgCdTe photodiode illuminated with 4.5 *μ*m laser pulses. The circle points are the experiment results, and the dash line is the fitting curve obtained by using the theory model. The dot line and dash dot line present the simulation of *pn* and Schottky potential PV response, respectively.

The dash line in Figure 5 is the best that fits with (1). The excellent fittings suggest a good reliability of the combined theory model of *pn* junction and Schottky barrier. The PV response curves of *pn* and Schottky barrier are also simulated respectively. The characteristic parameters values of transient photo-response are extracted from the fitting procedure. It can be seen that the response extremum, pulse rise time and fall time of the photovoltage generated by the Schottky contact are −68 mV, 3.3 ns and 20 ns, respectively, while they are 82 mV, 10 ns and 80 ns in *pn* junction. This shows that the pulse PV response generated by the Schottky barrier is negative and has a higher frequency characteristic respect to the *pn* junction ones. Thus the photo-response shows an apparent negative valley first, then it evolves into a positive peak. On the other hand, the negative PV response induced by the Schottky barrier will decrease the positive signal generated by *pn* junction, consequently reducing the response rate. As can be seen, although the simulation PV peak shown in Figure 5 is 55 mV, the experimental PV peak is partially offset by the negative PV induced by Schottky contact to only 39 mV, reducing the response rate to less than 70% of the simulation PV peak. If the performance of the electrode is poor, a larger Schottky barrier high between metal and the *p*-HgCdTe layers will exist at the interface. Thus, the negative PV will be more significant, even stronger than the positive PV. This is the reason why some HgCdTe photovoltaic devices appear to be anomalies negative open circuit voltage and the negative PV in the *I*-*V* test [13–15].

## 4. Conclusion

In summary, we have reported the effect of metal-semiconductor contact on the transient photovoltaic characteristic of HgCdTe PV detector. The Schottky barrier at the M/S interface cause the photo-response shows an apparent negative valley first, then it evolves into a positive peak. By employing a combined theoretical model of *pn* junction and Schottky potential, the characteristic parameters of transient photo-response are extracted. Utilizing this TPV characteristic of device, we can evaluate the electrode quality at the interface of metal/HgCdTe. Compared with the conventional *I*-*V* test, it can distinguish the characteristic parameter from *pn* junction. Thus, our initial result on the effect of M/S would make such TPV test a potential candidate for electrode assessment methods.
